# Pulmonary artery catheter versus pulse contour analysis: a prospective epidemiological study

**DOI:** 10.1186/cc5126

**Published:** 2006-12-14

**Authors:** Shigehiko Uchino, Rinaldo Bellomo, Hiroshi Morimatsu, Makoto Sugihara, Craig French, Dianne Stephens, Julia Wendon, Patrick Honore, John Mulder, Andrew Turner, the PAC/PiCCO Use and Likelihood of Success Evaluation [PULSE] Study Group

**Affiliations:** 1 Department of Emergency and Critical Care Medicine, Saitama Medical Center, 1981 Tsujido-machi, Kamoda, Kawagoe-shi, Saitama, 350-8550, Japan; 2 Department of Intensive Care and Department of Medicine, Austin Hospital, Studley Road, Heidelberg, Melbourne, 3084, Australia; 3 Department of Anesthesiology and Resuscitology, Okayama University Medical School, 2-5-1, Shikatacho, Okayama, 700-8558, Japan; 4 Tertiary Emergency Medical Center, Tokyo Metropolitan Bokuto Hospital, 4-23-15, Kotobashi, Sumidaku, Tokyo, 130-8575, Japan; 5 Western Hospital, Gordon Street Footscray, Melbourne, Melbourne, 3011, Australia; 6 Royal Darwin Hospital, Rocklands Drive, Tiwi, NT 0810, Australia; 7 King's College Hospital, Denmark Hill, London, SE 9RS, UK; 8 Departement de Medecine Aigue, Clinique Para-Universitaire St. Pierre, 9 Avenue Reine Fabiola, Ottignies-Louvain-La-Neuve, 1340, Belgium; 9 Epworth Hospital, 89 Bridge Road, Richmond, Melbourne, 3121, Australia; 10 Royal Hobart Hospital, 48 Liverpool St, Hobart, Tasmania, 7001, Australia

## Abstract

**Introduction:**

The choice of invasive systemic haemodynamic monitoring in critically ill patients remains controversial as no multicentre comparative clinical data exist. Accordingly, we sought to study and compare the features and outcomes of patients who receive haemodynamic monitoring with either the pulmonary artery catheter (PAC) or pulse contour cardiac output (PiCCO) technology.

**Methods:**

We conducted a prospective multicentre, multinational epidemiological study in a cohort of 331 critically ill patients who received haemodynamic monitoring by PAC or PiCCO according to physician preference in intensive care units (ICUs) of eight hospitals in four countries. We collected data on haemodynamics, demographic features, daily fluid balance, mechanical ventilation days, ICU days, hospital days, and hospital mortality. We statistically compared the two techniques.

**Results:**

Three hundred and forty-two catheters (PiCCO 192 and PAC 150) were inserted in 331 patients. On direct comparison, patients with PAC were older (68 versus 64 years of age; *p *= 0.0037), were given inotropic drugs more frequently (37.3% versus 13%; *p *< 0.0001), and had a lower cardiac index (2.6 versus 3.2 litres/minute per square meter; *p *< 0.0001). Mean daily fluid balance was significantly greater during PiCCO monitoring (+659 versus +350 ml/day; *p *= 0.017) and mechanical ventilation-free days were fewer (12 for PiCCO versus 21 for PAC; *p *= 0.045). However, after multiple regression analysis, we found no significant effect of monitoring technique on mean daily fluid balance, mechanical ventilation-free days, ICU-free days, or hospital mortality. A secondary multiple logistic regression analysis for hospital mortality which included mean daily fluid balance showed that positive fluid balance was a significant predictor of hospital mortality (odds ratio = 1.0002 for each ml/day; *p *= 0.0073).

**Conclusion:**

On direct comparison, the use of PiCCO was associated with a greater positive fluid balance and fewer ventilator-free days. After correction for confounding factors, the choice of monitoring did not influence major outcomes, whereas a positive fluid balance was a significant independent predictor of outcome. Future studies may best be targeted at understanding the effect of pursuing different fluid balance regimens rather than monitoring techniques *per se*.

## Introduction

The pulmonary artery catheter (PAC) has been a major haemodynamic monitoring tool in intensive care medicine for more than 30 years [1]. In haemodynamically unstable patients, the PAC might facilitate management and improve outcome. However, this view has been challenged by several observational and randomised controlled studies [2-4]. These studies suggest that (a) the information obtained is not useful; (b) due to misinterpretation, the information obtained is not used correctly; or (c) even if the information is useful and used correctly, overall patient outcome is determined by other processes that cannot be affected by haemodynamic monitoring and associated manipulations of the circulation.

More recently, new technology (PiCCO [pulse contour cardiac output] System; PULSION Medical Systems AG, Munich, Germany) that provides an alternative to the PAC has been developed and applied [5]. This new technology uses transpulmonary thermodilution and pulse contour analysis to calculate cardiac output, stroke volume variation, intra-thoracic blood volume, and extra-vascular lung water (EVLW). In patients who already have a central line, PiCCO requires only the insertion of a 4-French femoral catheter. Several small studies have been conducted to compare the PAC to PiCCO in terms of physiological relevance (for example, ability to predict fluid responsiveness). They have suggested that PiCCO-obtained data such as stroke volume variation or intra-thoracic blood volume index (ITBI) may better predict fluid responsiveness [5-10]. This may or may not affect clinical outcome.

Despite these physiological observations, very few studies have examined the overriding issue of clinical effectiveness [11]. The ideal way of testing the effectiveness of PiCCO would be by means of a randomised controlled trial. However, the cost of such a trial could be justified only if preliminary evidence suggested that PiCCO technology might provide clinically meaningful advantages or differences compared with PAC. Such preliminary evidence might be provided initially by evidence of a statistical association between PiCCO monitoring and better outcomes. Accordingly, we conducted a multicentre prospective epidemiological study to test the hypothesis that a significant association between the use of PiCCO and improved clinically relevant outcomes exists which would justify a subsequent randomised controlled trial.

## Materials and methods

This study was conducted in eight intensive care units (ICUs) in four countries (five in Australia, one in the United Kingdom, one in Belgium, and one in Japan) from March 2003 to April 2004. Because of the anonymous and non-interventional fashion of this study, ethical committees in all centres waived the need for informed consent.

### Study population

Patients were included in this study if they had a PiCCO catheter or PAC inserted while in the ICU. The only exclusion criteria were (a) PiCCO or PAC inserted outside the ICU (for example, operating room), (b) use of extracorporeal membrane oxygenation, or (c) use of a ventricular assist device. The exclusion of patients with a catheter inserted outside the ICU was based on the fact that no or very few centres currently have PiCCO insertion in the operating theatres, thus all elective patients or cardiac surgery patients would have had a PAC, creating a strong bias toward low mortality and short duration of mechanical ventilation in the population under study. All study patients were followed until hospital discharge.

### Data collection

Data collection was conducted by means of an electronically prepared Excel-based (Microsoft Corporation, Redmond, WA, USA) data collection tool. All centres were asked to complete data entry and to e-mail the data to the central office. On arrival, all data were screened in detail by a dedicated intensive care specialist for any missing information, logical errors, insufficient detail, or any other queries. Any queries generated an immediate e-mail inquiry with planned resolution within 48 hours.

The following information was prospectively obtained: gender, date of birth, dates of hospital and ICU admission, co-morbidities and pre-morbid renal function, SAPS II (simplified acute physiology score) [12] on the day of ICU admission, diagnosis, type of catheter inserted (PiCCO or PAC), dates of catheter insertion and removal, days of mechanical ventilation, ICU and hospital survival, and dates of ICU and hospital discharge. PiCCO- and PAC-specific variables (ITBI, extra-lung water index [ELWI], and pulmonary artery occlusion pressure [PAOP]) were also obtained at insertion. Reasons for catheter requirement were based on the judgement of the treating clinician. Because catheters were inserted to diagnose the cause of shock or hypoxia on some occasions, more than one reason could be chosen. Daily fluid balance data were also collected for 7 days or until catheter removal.

Co-morbidities (ischaemic heart disease [IHD], chronic obstructive pulmonary disease [COPD], and diabetes) were defined as follows. IHD was defined as a past history of acute myocardial infarction or coronary re-vascularisation. COPD was defined as documented abnormal lung function tests. Diabetes was defined as clinically previously diagnosed diabetes requiring medication (oral anti-hyperglycaemic or insulin). Previous renal function was defined as impaired if there was any evidence of abnormal renal function (high serum creatinine or low creatinine clearance) prior to hospital admission. End-stage renal failure was defined as present if a patient was on chronic dialysis. Previous renal function for which no information was available was labelled as unknown.

### Statistics

The primary hypothesis was that the length of ICU stay would be significantly shorter in patients managed by PiCCO than by PAC. We assumed, using published information [4], that the mean length of ICU stay in patients managed by PAC in ICU would be ten days with a standard deviation of eight days. Thus, 500 patients would be required for this study to have an 80% power of detecting a relative reduction of 20% in the mean length of ICU stay at an alpha of 0.05. We projected that we would be able to complete the study in six months. However, due to the withdrawal of trial units and slower-than-planned recruitment, we had reached only 300 patients after one year of data collection. Thus, we chose to conduct an interim analysis to test whether continued data collection was justified. At the interim analysis (300 patients), the unadjusted mean duration of ICU stay was 10.5 ± 10.7 days for PAC patients compared with 9.8 ± 10.3 days for PiCCO patients. Because of such a minor difference and the greater-than-expected standard deviation, we calculated that we would have required 2,729 patients in each arm for the study to have an 80% power to detect statistical significance at an alpha of 0.05. Accordingly, on the grounds of futility, we stopped recruitment.

Data are presented as medians (with 25th and 75th percentiles) or as percentages. The Fisher's exact test and Mann-Whitney test were used for nominal values and numerical variables, respectively, to compare variables in patients managed with PiCCO and PAC. A *p *value of less than 0.05 was considered statistically significant.

Multiple linear regression analysis was used to identify predictors for daily fluid balance, mechanical ventilation-free days, and ICU-free days at 28 days. Multiple logistic regression analysis was used for hospital mortality. All variables presented in Tables 1 and 2, except ITBI, ELWI, and PAOP, were chosen as independent variables in the analyses. A backward stepwise elimination process was used to remove variables that had a *p *value greater than 0.05. Use of PiCCO was forced to remain in the models. As a secondary process that was not part of the original data analysis plan, the analysis for hospital mortality was repeated with mean daily fluid balance included as an independent variable. A box plot graph was used to show daily fluid balance from days one to seven. The StatView statistical package (Abacus Concepts, Inc., Berkeley, CA, USA) was used for the above statistical analyses.

## Results

Three hundred and forty-two catheters (PiCCO 192 and PAC 150) were inserted in 331 patients. Eleven patients had both PiCCO and PAC either at the same time or sequentially. These patients were excluded from multiple regression analyses. During this study, two centres were found to have used PAC monitoring exclusively and one to have used PiCCO monitoring exclusively. The other five centres were found to have used both techniques (Table 3).

Demographics of patients are shown in Table 1. Compared with patients with PiCCO, patients with PAC were older (68 versus 64 years; *p *= 0.0037), were more likely to have received inotropes (37.3% versus 13.0%; *p *< 0.0001), had a lower cardiac index (2.6 versus 3.2 litres/minute per square millimetre; *p *< 0.0001), and were less likely to be on renal replacement therapy (16.7% versus 26.6%; *p *< 0.0001) at recruitment. Diagnostic groups and reasons for catheter insertion are shown in Table 2.

The most common diagnostic group was cardiac disease; approximately 60% of patients with a PAC were in this group. Although the cardiac diagnostic group was also the most common group in patients with PiCCO, respiratory, gastrointestinal, and hepatic conditions were also common. Septic shock was the most common cause of catheter insertion in patients with PiCCO, and cardiogenic shock was the most common cause in patients with PAC.

Suspected combined cardiogenic and septic shock was chosen by the treating clinicians as the reason for insertion for 39 catheters (27 PiCCO and 12 PAC), with the catheter being inserted to help diagnose the cause of shock. Similarly, both fluid overload and acute respiratory distress syndrome/acute lung injury (ALI) were chosen as justification for the insertion of six catheters (four PiCCO and two PAC), with the catheter used to help diagnose the cause of lung dysfunction.

**Figure 1:**
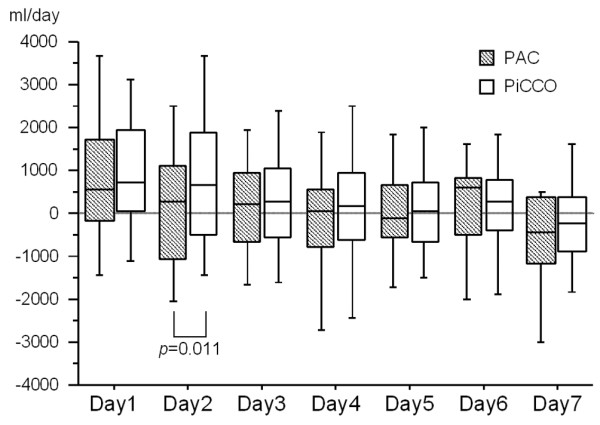
Box plot diagram illustrating daily fluid balance during seven days of invasive monitoring. During day two, fluid balance was more positive in PiCCO-monitored patients compared with PAC-monitored patients (*p *= 0.011). PAC, pulmonary artery catheter; PiCCO, pulse contour cardiac output.

Daily fluid balance is shown in Figure 1. On unadjusted comparison, patients with PiCCO tended to have a more positive fluid balance compared with patients with PAC and fluid balance was found to be significantly different on day two.

Patient outcomes are shown in Table 4. Unadjusted mean daily fluid balance was significantly greater with PiCCO. Mechanical ventilation-free days were fewer with PiCCO. All other outcomes, including ICU days, also tended to be worse in PiCCO-monitored patients.

Because we expected the demographic and clinical features of the two groups to be different, multiple regression analysis had been planned and was accordingly conducted to adjust and compare the impact of catheter choice on mean daily fluid balance, mechanical ventilation-free days, ICU-free days, and hospital mortality (Tables 5, 6, 7, 8). None of these analyses showed choice of PiCCO as the monitoring technique to be a significant independent predictor of these clinical outcomes.

A secondary multiple regression analysis was repeated for hospital mortality, with mean daily fluid balance included after initial analysis suggested a strong fluid balance-related effect. This secondary analysis showed that a positive fluid balance was a significant independent predictor of hospital mortality.

Further analysis was conducted including only the five centres that had used both techniques during the study. All findings remained statistically equivalent to those seen with the entire cohort.

In all multivariate logistic regression analyses, we also sought to assess the role of multicollinearity. The variance inflation factor was calculated for each variable in the final model and was found to be less than 5, consistent with a lack of severe multicollinearity.

## Discussion

We conducted a multicentre, multinational, prospective epidemiological study of the clinical use of PICCO catheters and PACs in more than 300 patients to study whether catheter selection showed an independent association with clinical outcomes. We found that the two catheters were applied to different populations, with the PAC preferentially applied to patients with cardiac conditions and PiCCO preferentially applied to patients with septic shock or other non-cardiac conditions. On direct univariate comparison, we found that the use of PiCCO was associated with a more positive fluid balance and fewer mechanical ventilation-free days. However, after planned statistical correction for differences in patient features, the use of either catheter was not associated with any clinical advantage or disadvantage. On secondary analysis and after similar statistical corrections, we also found that a positive fluid balance was a predictor of increased mortality.

PiCCO is a recently developed transpulmonary thermo-dilution technique for invasive haemodynamic monitoring, with which not only continuous cardiac output, but also several volume-related variables can be obtained [5]. Several studies have repeatedly shown that PiCCO-derived indices can more accurately predict increases in cardiac output with fluid resuscitation [6-10]. PiCCO is becoming popular for the management of critically ill patients [13].

However, few studies have examined the clinical effectiveness of PiCCO or transpulmonary dilution techniques. Sakka and colleagues. [14] retrospectively analysed 373 critically ill patients managed with transpulmonary thermo-dye dilution technique and found that EVLW was a significant predictor of mortality, but no comparative group was included in this study. Only one study has compared the transpulmonary dilution technique with the PAC [11]. In that study, 52 patients were randomly assigned to an EVLW management group and 49 patients to a PAOP-based management group. Fluid management was conducted differently, as evidenced by a median cumulative fluid balance of 754 ml in the EVLW group versus 1,600 ml in the PAC group (*p *= 0.001). Ventilator days and ICU days were significantly shorter in the EVLW group. Although its results were provocative, it was a single-centre study and no further trial has subsequently confirmed this finding. Furthermore, the technology used was not the current single-injection technology. Finally, fluid management based on PAOP is not a widely accepted approach and is open to much criticism on physiological grounds. To further explore whether a case might exist to justify a randomised controlled trial comparing these techniques, we conducted a multicentre prospective observational study comparing the clinical use of PiCCO and PAC and their independent association with several relevant clinical outcomes.

Not surprisingly, PAC was used more often for patients with cardiac disorders than PiCCO. Patients with PAC were also older, had a lower blood pressure and cardiac index, and were more likely to have been treated with inotropes compared with patients with PiCCO. Univariate analysis showed that patients with PiCCO had significantly fewer mechanical ventilation-free days and had a more positive fluid balance. However, planned multiple regression analysis found that the choice of monitoring technique was not a significant predictor of outcome.

Patients managed with PiCCO were given more fluid in most of the observation period, although the difference in fluid became statistically significant only on day two. The reason for this difference may lie in the effect of the monitoring technique itself. The perception of a greater ability to predict fluid responsiveness [6-10] once PiCCO was applied might have induced a greater number of fluid challenges or more aggressive fluid challenges. Alternatively, the difference might reflect the fact that more cardiac failure patients received PAC monitoring and that such patients elicited a more negative fluid balance to deal with the presence of pulmonary oedema.

Less fluid given to critically ill patients has been shown to be related to better outcomes in various situations (for example, septic shock [15], pulmonary oedema [16], abdominal compartment syndrome [17], trauma and haemorrhagic shock [18], hepatectomy [19], and colonic resection [20]). Consistent with these studies, on secondary (*post hoc*) analysis, we found that a positive mean daily fluid balance was a significant predictor of hospital mortality in multiple logistic regression analysis (*p *= 0.0073). Once mean daily fluid balance was included in the multiple regression analysis for hospital mortality, the odds ratio for PiCCO was reduced (from 1.58 to 1.38).

Our study has several limitations. First, this is an observational study, not a randomised trial. Therefore, our findings could be explained by selection bias. However, data were collected from eight centres in four countries, and multiple analyses were conducted to correct for such biases, and the study was sufficiently powered to detect clinically meaningful associations. Association is a typical first step to justify subsequent randomised investigations of clinical effectiveness. As has been shown repeatedly (the higher the blood pressure with nitric oxide synthase inhibitor for septic shock, the lower the PCO_2 _[partial pressure of carbon dioxide] with high tidal volume for ALI), physiological efficacy is not always related to clinical effectiveness [21,22]. This might be the case with the transpulmonary dilution technique. Second, our aim was not to evaluate the impact of daily fluid balance on clinical outcome; this was a *post hoc *finding, which should be treated with much caution. Third, we excluded patients in whom the PAC or PiCCO was inserted in the operating room, such as cardiac surgery patients. In these patients, information obtained via PiCCO monitoring might have different effects [23-25]. However, these patients still mostly receive PAC monitoring and have favourable outcomes in 97% to 98% of cases with a short (<24 hours) ICU stay. Their inclusion would require a study of thousands of patients. Fourth, the concept of adjusting data analysis may be fundamentally flawed despite attempts to statistically define independent explanatory variables. The quantification of the impact of such variables and interventions, which are likely to result from the use of the selected monitoring strategy or to reflect the information the technology provides, may not be controllable with multivariate regression. For example, if haemodynamic monitoring is used to guide fluid administration, the use of fluid balance (as a continuous variable over the duration of the study) or the use of vasoactive drugs (a categorical variable at study inclusion) as independent variables may well fail to assess the complex associations between the information obtained from the monitoring technology and the administration of fluids or vasoactive drugs over the seven-day period of data collection. Furthermore, potential explanatory variables may not have the linear characteristics assumed for such analysis. Finally, other variables may have existed (unknown or incorrectly omitted) which powerfully influenced outcome but which were not included in the models. These important confounders must be taken into account when assessing the findings of our study.

## Conclusion

We have conducted a multicentre, multinational epidemiological study of invasive haemodynamic monitoring in ICU. On direct comparison, we found that the use of PiCCO was associated with a greater positive fluid balance and fewer ventilator-free days. After correction for confounding factors by multiple regression analysis, the choice of technique for invasive haemodynamic monitoring did not appear to influence major outcomes in critically ill patients. Furthermore, on *post hoc *analysis, we found that a positive fluid balance was a significant independent predictor of outcome. Future studies may best be targeted at understanding the effect of pursuing different fluid balance regimens rather than monitoring techniques *per se*.

## Key messages

• We conducted the first multicentre, multinational epidemiological study comparing the use of PiCCO and PAC in the ICU.

• In this study, invasive haemodynamic monitoring with PiCCO or PAC appeared to be applied to different patient populations.

• PiCCO was preferentially used in patients with vasodilatory shock, whereas PAC was more commonly applied to patients requiring inotropic support.

• On direct comparison, the use of PiCCO was associated with a greater positive fluid balance and fewer ventilator-free days.

• Once adjustment was made for confounding variables, the choice of monitoring technique did not predict outcome, but a positive fluid balance was a significant predictor of greater mortality.

## Abbreviations

ALI = acute lung injury; COPD = chronic obstructive pulmonary disease; ELWI = extra-lung water index; EVLW = extra-vascular lung water; ICU = intensive care unit; IHD = ischaemic heart disease; ITBI = intra-thoracic blood volume index; PAC = pulmonary artery catheter; PAOP = pulmonary artery occlusion pressure; PiCCO = pulse contour cardiac output.

## Competing interests

One of the PULSE investigators (JW) is a member of a Medical Advisory Board for PULSION Medical Systems AG, the company that makes and markets the PiCCO monitoring device.

## Authors' contributions

SU and RB conceived and designed the study and wrote the manuscript. SU performed the statistical analysis. HM, MS, CF, DS, JW, PH, JM, and AT assisted with the study design development and participated in its execution. All authors reviewed the manuscript and contributed to its final version. All authors read and approved the final manuscript.

## Figures and Tables

**Table 1:** Demographic features of study of patients

	All patients (*n *= 331)	PiCCO (*n *= 192)	PAC (*n *= 150)	*p *value
At ICU admission				
Gender (male)	58.9%	57.3%	60.0%	0.66
Age in years	67 (54, 75)	64 (47, 74)	68 (57, 76)	0.0037
NYHA: III, IV	11.5%	10.9%	12.0%	0.86
IHD	31.1%	32.3%	28.7%	0.48
COPD	16.0%	14.1%	18.7%	0.30
Diabetes	22.1%	25.0%	19.3%	0.24
Previous renal function				
Normal	58.6%	59.4%	58.7%	0.91
Impaired	22.4%	22.4%	22.0%	>0.99
ESRF	3.9%	4.7%	2.7%	0.40
Unknown	15.1%	13.5%	16.7%	0.45
SAPS II	49 (37, 61)	49 (37, 61)	47 (37, 61)	0.91
At study inclusion				
Vasopressors	73.4%	75.0%	71.3%	0.46
Inotropic drugs	22.1%	13.0%	37.3%	<0.0001
Heart rate (beats per minute)	98 (84, 115)	99 (85, 118)	97 (84, 111)	0.48
MAP (mm Hg)	75 (68, 85)	76 (70, 85)	73 (63, 84)	0.0086
Cardiac index (litres/minute per m^2^)	3.0 (2.3, 4.0)	3.2 (2.6, 4.5)	2.6 (2.1, 3.5)	<0.0001
CVP (mm Hg)	12 (8, 15)	12 (9, 16)	11 (8, 14)	0.011
ITBI (ml/m^2^)	-	967 (768, 1,140)	-	
ELWI (ml/kg)	-	8.9 (6.6, 13.0)	-	
PAOP (mm Hg)	-	-	17 (12, 22)	
Mechanical ventilation	81.6%	80.2%	84.0%	0.40
PEEP (cm H_2_O)	5 (5, 10)	6 (5, 10)	5 (5, 8)	0.21
PaO_2_/FiO_2 _ratio (Torr)	186 (125, 279)	191 (126, 279)	185 (125, 285)	0.87
RRT	21.4%	26.6%	16.7%	0.036
Urea (mmol/l)	12.1 (7.1, 19.0)	12.0 (7.1, 18.7)	12.2 (7.1, 19.1)	0.91
Creatinine (μmol/l)	142 (100, 231)	151 (100, 231)	137 (97, 230)	0.42

Values are presented as medians (with 25th and 75th percentiles) or as percentages. COPD, chronic obstructive pulmonary disease; CVP, central venous pressure; ELWI, extra-vascular lung water index; ESRF, end-stage renal failure; ICU, intensive care unit; IHD, ischaemic heart disease; ITBI, intra-thoracic blood volume index; MAP, mean arterial pressure; NYHA, New York Heart Association; PAC, pulmonary artery catheter; PAOP, pulmonary artery occlusion pressure; PaO_2_/FiO_2_, partial pressure of oxygen in arterial blood/fraction of inspired oxygen; PEEP, positive end-expiratory pressure; PiCCO, pulse contour cardiac output; RRT, renal replacement therapy; SAPS II, simplified acute physiology score.

**Table 2:** Diagnostic groups and reasons for catheter insertion

	All	PiCCO	PAC	*p *value
Diagnostic groups				
Cardiac	41.4%	28.1%	60.7%	<0.0001
Respiratory	23.9%	26.6%	19.3%	0.12
Gastrointestinal	10.9%	12.5%	8.7%	0.30
Hepatic	10.0%	16.1%	2.0%	<0.0001
Renal	2.7%	2.1%	2.7%	0.73
Trauma	1.8%	2.6%	0.7%	0.18
Metabolic	1.5%	1.6%	1.3%	0.86
Others	7.9%	14.6%	6.7%	0.024
Reasons				
Septic shock	44.7%	51.6%	32.0%	0.0003
Cardiogenic shock	43.8%	36.5%	55.3%	0.0007
Other types of shock	13.3%	10.9%	15.3%	0.26
Fluid overload	19.6%	14.1%	25.3%	0.012
ARDS/ALI	6.0%	4.7%	7.3%	0.36
PE/PH	1.5%	0.0%	4.0%	0.0067
Other reasons	4.8%	7.8%	2.0%	0.026

More than one reason could be chosen to diagnose the cause of shock or hypoxemia. ALI, acute lung injury; ARDS, acute respiratory distress syndrome; PAC, pulmonary artery catheter; PE, pulmonary embolism; PH, pulmonary hypertension; PiCCO, pulse contour cardiac output.

**Table 3:** Characteristics and number of catheters in each centre

Centre	Country	Academic	Type of ICU	Patients	PAC	PiCCO
1	Australia	Yes	General	91	45	49
2	Japan	No	Emergency	36	36	0
3	Australia	No	General	61	18	47
4	UK	Yes	Liver	51	0	51
5	Belgium	Yes	General	26	26	0
6	Australia	Yes	General	19	4	16
7	Australia	No	General	4	1	3
8	Australia	Yes	General	43	20	26

Total				331	150	192

ICU, intensive care unit; PAC, pulmonary artery catheter; PiCCO, pulse contour cardiac output.

**Table 4:** Clinical outcomes for study patients according to monitoring tool

	All patients	PiCCO	PAC	*p *value
Mean daily FB (ml/day)	490 (-216, 1,259)	659 (-128, 1,403)	350 (-573, 1,064)	0.017
MV days	5 (2, 10)	6 (2, 10)	4 (2, 10)	0.44
MV-free days in 28 days	15 (0, 24)	12 (0, 23)	21 (0, 25)	0.045
ICU days	6 (3, 14)	7 (3, 13)	6 (3, 14)	0.58
ICU-free days in 28 days	11 (0, 22)	5 (0, 21)	14 (0, 22)	0.15
Hospital days	19 (9, 38)	20 (9, 45)	17 (8, 33)	0.15
ICU mortality	34.7%	38.5%	30.0%	0.11
Hospital mortality	42.0%	45.8%	36.9%	0.12

Values are presented as medians (with 25th and 75th percentiles) or as percentages. FB, fluid balance; ICU, intensive care unit; MV, mechanical ventilation; PAC, pulmonary artery catheter; PiCCO, pulse contour cardiac output.

**Table 5:** Multiple linear regression analysis with mean daily fluid balance (ml/day) as the dependent outcome variable

	Coefficient (95% CI)	*p *value
R: Other types of shock	1,027 (522, 1,532)	<0.0001
R: Fluid overload	-770 (-1,210, -331)	0.0006
SAPS II (per point)	15.5 (5.8, 25.1)	0.0018
Inotropes	-536 (-954, -119)	0.012
Heart rate (beats per minute)	9.8 (2.2, 17.5)	0.012
Dx: Others	713 (76, 1,351)	0.028
COPD	475 (27, 923)	0.038
CVP	-31.9 (-62.1, -1.6)	0.039
Creatinine (μmol/l)	-1.3 (-2.6, -0.0)	0.044
PiCCO	209 (-147, 564)	0.25

Coefficient values are presented as medians (with 25th and 75th percentiles). R^2 ^= 0.237. CI, confidence interval; COPD, chronic obstructive pulmonary disease; CVP, central venous pressure; Dx, diagnostic group; PiCCO, pulse contour cardiac output; R, reason for catheter insertion; SAPS II, simplified acute physiology score.

**Table 6:** Multiple linear regression analysis with mechanical ventilation-free days as the dependent outcome variable

	Coefficient (95% CI)	*p *value
SAPS II (per point)	-0.18 (-0.24, -0.11)	<0.0001
R: Fluid overload	5.76 (2.95, 8.57)	<0.0001
PEEP (cm H_2_O)	-0.47 (-0.78, -0.17)	0.0026
Age (per year)	-0.12 (-0.20, -0.04)	0.0027
PaO_2_/FiO_2 _ratio (Torr)	0.01 (0.00, 0.02)	0.014
RRT	-3.11 (-5.95, -0.27)	0.032
R: Cardiogenic shock	2.44 (0.11, 4.77)	0.041
PiCCO	-1.51 (-3.82, 0.80)	0.20

Coefficient values are presented as medians (with 25th and 75th percentiles). R^2 ^= 0.259. CI, confidence interval; PaO_2_/FiO_2_, partial pressure of oxygen in arterial blood/fraction of inspired oxygen; PEEP, positive end-expiratory pressure; PiCCO, pulse contour cardiac output; R, reason for catheter insertion; RRT, renal replacement therapy; SAPS II, simplified acute physiology score.

**Table 7:** Multiple linear regression analysis with ICU-free days as the dependent outcome variable

	Coefficient (95% CI)	*p *value
SAPS II (per point)	-0.19 (-0.25, -0.13)	<0.0001
R: Fluid overload	4.92 (2.31, 7.52)	0.0002
R: Cardiogenic shock	3.85 (1.72, 5.97)	0.0004
RRT	-4.49 (-7.13, -1.85)	0.0009
Vasopressors	-3.51 (-5.89, -1.13)	0.004
Dx: Renal	8.25 (2.02, 14.47)	0.0096
Age (per year)	-0.08 (-0.15, -0.01)	0.035
PaO_2_/FiO_2 _ratio (Torr)	0.01 (0.00, 0.02)	0.039
PiCCO	-0.49 (-2.62, 1.64)	0.65

Coefficient values are presented as medians (with 25th and 75th percentiles). R^2 ^= 0.306. CI, confidence interval; Dx, diagnostic group; ICU, intensive care unit; PaO_2_/FiO_2_, partial pressure of oxygen in arterial blood/fraction of inspired oxygen; PiCCO, pulse contour cardiac output; R, reason for catheter insertion; RRT, renal replacement therapy; SAPS II, simplified acute physiology score.

**Table 8:** Multiple logistic regression analysis with hospital mortality as the dependent outcome variable

	Odds ratio (95% CI)	*p *value
Without FB		
Age (per year)	1.04 (1.02, 1.06)	<0.0001
SAPS II (per point)	1.05 (1.03, 1.06)	<0.0001
RRT	2.41 (1.23, 4.73)	0.010
Dx: Renal	0.08 (0.01, 0.58)	0.012
R: Fluid overload	0.44 (0.23, 0.88)	0.019
Dx: Respiratory	1.93 (1.06, 3.53)	0.033
PiCCO	1.58 (0.92, 2.71)	0.10
With FB		
Age (per year)	1.04 (1.02, 1.06)	<0.0001
SAPS II (per point)	1.04 (1.02, 1.06)	<0.0001
RRT	2.53 (1.24, 5.16)	0.011
Dx: Renal	0.47 (0.22, 0.98)	0.044
R: Fluid overload	0.08 (0.01, 0.7)	0.022
Mean daily FB (ml/day)	1.0002 (1.0001, 1.0004)	0.0073
PiCCO	1.38 (0.78, 2.44)	0.27

Odds ratios are presented as medians (with 25th and 75th percentiles). R^2 ^= 0.205 without FB and 0.191 with FB. CI, confidence interval; Dx, diagnostic group; FB, fluid balance; PiCCO, pulse contour cardiac output; R, reason for catheter insertion; RRT, renal replacement therapy; SAPS II, simplified acute physiology score.
